# Vitamin A at the interface of host–commensal–pathogen interactions

**DOI:** 10.1371/journal.ppat.1007750

**Published:** 2019-06-06

**Authors:** Namrata Iyer, Shipra Vaishnava

**Affiliations:** Department of Molecular Microbiology and Immunology, Brown University, Providence, Rhode Island, United States of America; Nanyang Technological University, SINGAPORE

## Introduction

The gut is a stable ecosystem governed by interactions between the host immune system and trillions of gut-resident microbes. Dietary vitamin A has long been identified as the anti-infective agent that plays a key role in regulating multiple aspects of the gut immune system. Recent studies show that both commensal and pathogenic bacteria drastically alter vitamin A metabolism in the host. How microbial regulation of vitamin A metabolism influences enteric infections is not fully understood. Elucidating the molecular mechanisms underlying vitamin A metabolism during healthy and diseased states is critical for designing optimal nutritional therapies to treat infectious disease. Here, we discuss how the complex interplay between dietary vitamin A, mucosal immunity, and commensal bacteria influences the outcome of enteric infections.

## Vitamin A shapes host–microbe interactions in the gut

Vitamin A is an essential micronutrient derived from the diet in the form of carotenoids and retinyl esters. Upon absorption by epithelial cells in the intestine, these carotenoids are sequentially converted into retinol and then the active form, retinoic acid (RA), or the liver storage form, retinyl esters. RA drives transcription programs within cells, which are vital for cell fate determination in the embryo and for the development of the immune system [1].

Vitamin A is central to immune homeostasis in the gut, coordinating both innate and adaptive immunity. RA stimulates the migration of immune cells including dendritic cells, T cells, and B cells to the intestine and helps inform their function [2–4]. Vitamin A directly regulates proliferation and differentiation in the intestinal epithelium, which is crucial to the maintenance of the gut barrier [5].

The effect of vitamin A is not limited to the host. Acute vitamin A deficiency triggers a significant change in the community structure in human gut microbiota–associated mice [6]. RA regulates the levels of antimicrobials as well as secretory immunoglobulin A (IgA), which also influences the microbial composition in the gut [7, 8]. A recent study has discovered synthetic retinoids that have direct antibacterial properties, adding a new dimension to vitamin A–microbiome interactions [9].

## Microbial regulation of vitamin A metabolism

The potency of RA necessitates that its synthesis be tightly controlled. A suite of metabolic enzymes determine how much vitamin A becomes RA and how much is sent to the liver for storage. Intestinal epithelial cells (IECs) as well as dendritic cells express the vitamin A metabolic machinery; however, not much is known about its regulation. Recently, the gut microbiome has been identified as a new player in host vitamin A metabolism. Germ-free mice express higher levels of RA-synthesizing enzymes in their intestinal epithelium than mice with a regular microbiome express. Colonization of the gut by microbes reduces RA levels in the intestine, which is balanced by an increase in the liver storage form. This regulation appears to be dynamic and microbe specific. Spore-forming bacteria such as *Clostridia* promote the reduction in RA levels, whereas dysbiotic microbial communities have the opposite effect [10]. This is a novel feedback loop in the gut wherein vitamin A modulates the microbiome, which in turn tunes host immunity via vitamin A metabolism in the gut.

## Altered vitamin A status during infection

**Fig 1:**
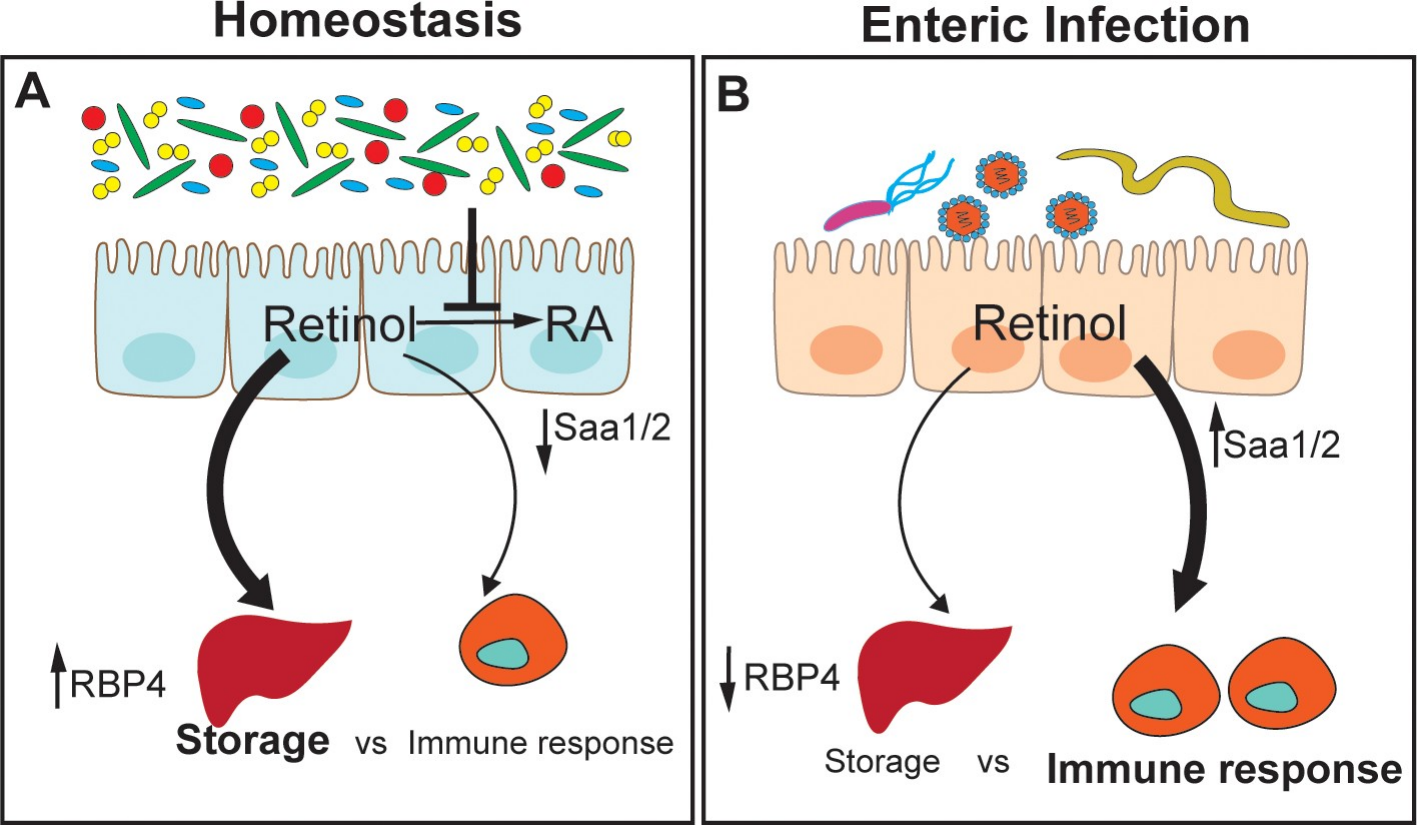
Vitamin A (retinol) handling during health and disease. (A) During homeostasis, gut bacteria suppress the conversion of dietary retinol into RA and promote its storage. Retinol transporter RBP4 is the main transporter of retinol to and from the liver. (B) During infection, RBP4 amounts drop, reducing retinol transport and storage in the liver. Acute-phase RBPs, such as SAAs, increase in the intestinal tissue and up-regulate local immune response to infection. RA, retinoic acid; RBP, retinol-binding protein; SAA, serum amyloid A.

The first response to infection involves rapid and dramatic changes in protein synthesis of acute-phase proteins [11]. Both retinol-binding protein (RBP) and transthyretin, which transport retinol, decline during acute-phase response to the infection, resulting in undetectable levels of serum retinol. Low serum retinol, i.e., hyporetinolemia, has been reported in children and adults in association with acute infections (e.g., measles, malaria, diarrhea, HIV), multiple morbidities, and trauma. It has been established that retinol and RBP concentrations are inversely correlated with serum concentrations of interleukin-6. This cytokine is the major regulator of the acute-phase response and induces the gene expression of many acute-phase proteins [12]. In animal models in which acute-phase response was induced by injecting lipopolysaccharide (LPS), it was noted that serum hyporetinolemia is caused by a reduction in liver RBP synthesis initiated by the reduced transcription of *Rbp4* [13]. In contrast to reduction in RBP4 during infection, alternate acute-phase proteins called serum amyloid A (SAA1 and 2) that bind retinol with high affinity are strongly up-regulated in the intestinal as well as liver tissues [14]. SAAs are required to generate T helper 17 (Th17) cells that modulate antimicrobial production and thus microbial composition in the gut [15]. Up-regulation of SAAs during microbial infection suggests that the host engages an alternate vitamin A mobilization machinery to mount a specific antimicrobial response (Fig 1).

### Vitamin A status and colonization resistance to enteric pathogens

The ability of an invading pathogen to cause disease in the gut depends on 2 factors: (1) effective competition with the commensals for nutrition and space and (2) evasion of host immune response. Dietary vitamin A status has a significant impact on homeostatic host–microbiome interactions and thus susceptibility to pathogen invasion. Recent studies have helped shed light on the mechanisms by which vitamin A affects host resistance to infections.

### Enterobacterial infections

Enterobacteria contribute to almost a third of diarrheal infections in children across the globe. These gram-negative, facultative anaerobic microbes employ a variety of strategies for pathogenesis. Enteropathogenic *Escherichia coli* (EPEC) and enterohemorrhagic *E*. *coli* (EHEC) use attachment to the intestinal epithelium to disrupt the gut barrier. Others, such as *Salmonella*, invade the epithelial cells and trigger an inflammatory cascade that leads to pathology. Vitamin A deficiency is linked to increased susceptibility to enteric infections. Recently, some studies have shed more light on the role of vitamin A during enterobacterial infections.

*Citrobacter rodentium* is a murine pathogen that shares virulence mechanisms with EPEC. Vitamin A–deficient (VAD) mice are more susceptible to *Citrobacter* infection. *Citrobacter* infection, although usually self-limiting, can cause almost 40% mortality in VAD mice. Deficient mice that do survive are unable to clear the infection and become chronically infected with the pathogen [16]. An important cytokine required to clear the pathogen is interleukin-22 (IL-22). Produced by Th17 and type 3 innate lymphoid cells (ILC3), IL-22 regulates the production of antimicrobials such as regenerating islet-derived protein 3 gamma (Reg3γ) and calprotectin in the gut. RA affects the numbers of Th17 and ILC3 recruited into the gut as well as the expression of IL-22 itself. The lack of vitamin A and thus IL-22 is responsible for the higher susceptibility of VAD mice to *Citrobacter* [17]. Interestingly, *Citrobacter* infection in vitamin A–sufficient mice induces a depletion of liver reserves of vitamin A, whereas intestinal and serum retinol are unaffected. Liver retinol levels were lower than that seen with a lethal lung infection and did not recover even after *Citrobacter* clearance. This implies that infections in the gut, the site of vitamin A absorption, can take a significant toll on vitamin A liver stores in the body, even if the infection is successfully cleared [18]. Whether such depletion of liver stores of vitamin A can temporarily impair the host’s ability to fight off successive enteric infections is currently unknown.

Vitamin A has an unexpected effect on EHEC infections. Deficiency in vitamin A worsens the intestinal damage caused by EHEC; however, host survival sees an overall improvement. Lethality in EHEC infections is associated with Shiga toxin production. Shiga toxin produced in the intestine can sometimes enter the circulation, leading to fatal hemolytic syndrome. The potency of the toxin is increased by the reactive oxygen species (ROS) produced by polymorphonuclear cells (PMNs). Vitamin A deficiency, although detrimental in the intestine, results in less recruitment of PMNs and reduced ROS. The increased survival in VAD mice is attributed to ineffective activation of the Shiga toxin [19].

Vitamin A deficiency is classically known to increase susceptibility to infections by nontyphoidal *Salmonella* serovars. Counterintuitively, too much RA in the gut can aid colonization by the pathogen. *Salmonella* Typhimurium depends on the inflammatory response by the host to gain an edge over gut commensals. During infection, the host increases IL-22–mediated Reg3γ and calprotectin production in the gut. Owing to its resistance to these antimicrobials, *Salmonella* blooms in the gut, whereas protective commensal species, such as *Clostridia*, perish. RA made by IECs promotes the IL-22 response. Intriguingly, commensals like *Clostridia* can reduce RA synthesis by IECs. Thus, by dampening RA synthesis, commensal species limit *Salmonella’s* ability to hijack the host immune response [10].

### Other enteric pathogens

Vitamin A deficiency leads to a depletion of IL-22–producing ILC3 in the gut. However, this blunted ILC3 response is accompanied by an increase in type 2 ILC (ILC2) response, which results in goblet cell hyperplasia and a thicker mucus barrier. This compensatory ILC2 response allows the intestine to successfully resist nematode infections and maintain barrier integrity [17]. The effects of vitamin A go beyond the intestine, affecting the number as well as function of resident macrophages in the liver. Macrophages are important for defense against helminth infections, and vitamin A deficiency results in increased mortality during infection by the trematode *Schistosoma mansoni* [20].

Norovirus is a notorious pathogen responsible for the majority of nonbacterial gastroenteritis cases in industrialized countries. The host relies on a T helper 1 response to thwart the infection. Clinical trials show that vitamin A supplementation significantly reduces the incidence of norovirus infection and progression to diarrhea but prolongs viral shedding in the feces [21]. A recent study in mice proposed a possible mechanism for this wherein RA favors the emergence of *Lactobacillus* sp., which promotes an interferon β (IFNβ) response and helps to inhibit viral replication [22].

## Future perspectives

Vitamin A is critical to many aspects of health, from embryonic development to immune homeostasis. In developing countries, vitamin A deficiency is a chronic problem that leads to high susceptibility to enteric infections in children. Maternal malnutrition is an underappreciated factor in infection susceptibility. Maternal vitamin A status influences immune development in the fetus [23]. Furthermore, the maternal microbiota is a prominent factor governing immune competency in the fetus, another aspect that could be affected by maternal vitamin A deficiency [24]. Surprisingly, irrespective of maternal vitamin A status, newborns have negligible stores of vitamin A in the liver. They rely on breast milk and other dietary factors to build liver stores. How stores of vitamin A in liver, serum, or tissues affect immune competency and infection susceptibility is an exciting area for future research. What we do know is that the relationship between vitamin A and infection is not black and white. Both too little and too much vitamin A can have negative consequences based on the infection context. Also, vitamin A metabolism in different tissue compartments can have differing effects on immunity and infection. In populations at risk for vitamin A deficiency, vitamin A supplementation in young children (<5 years old) significantly reduces the mortality associated with diarrhea. Interestingly, supplementation has no significant effect on diseases such as measles and respiratory illnesses [25]. This underscores the clinical benefits of interventions correcting vitamin A deficiency, especially for enteric diseases. A more comprehensive understanding of vitamin A metabolism and distribution in the body is required to better inform how we define vitamin A deficiency and help us design effective dosing and delivery strategies to reduce the morbidity associated with enteric diseases. Furthermore, probiotics can be mined for their ability to modulate vitamin A metabolism. A combination of probiotics and vitamin A supplements can be a holistic way to restore vitamin A sufficiency while maintaining host–commensal homeostasis.
